# Pleomorphic spindle cell sarcoma (PSCS) (formerly known as malignant fibrous histiocytoma, MFH) of the spleen, mimics an atypical haemangioma on ^99m^Tc-RBC, CT and Ultrasound: staging with ^18^F-FDG PET/CT

**DOI:** 10.1259/bjrcr.20150519

**Published:** 2017-03-30

**Authors:** William Makis, Karim Samji, Ryan W Hung, Jean Deschenes

**Affiliations:** ^1^Department of Diagnostic Imaging, Cross Cancer Institute, Edmonton, AB, Canada; ^2^Department of Diagnostic Imaging, University of Alberta Hospital, Edmonton, AB, Canada; ^3^Department of Pathology, Cross Cancer Institute, Edmonton, AB, Canada

## Abstract

A 63-year-old male was found to have a 7.5-cm splenic mass that had imaging appearances of an atypical haemangioma on CT, ultrasound and a ^99m^Tc-RBC scan, and he was followed conservatively with serial ultrasounds. Sixteen months later, however, the splenic lesion grew and he developed numerous new liver masses which were biopsy confirmed to be a pleomorphic spindle cell sarcoma (PSCS), formerly known as malignant fibrous histiocytoma (MFH). A staging ^18^F-FDG PET/CT was performed and showed innumerable, mostly necrotic hepatic and splenic masses. The patient passed away a few days after the PET/CT, before a treatment program could be implemented. The use of ^18^F-FDG PET/CT in the staging of splenic PSCS has not been previously described. We present the ^99m^Tc-RBC and ^18^F-FDG PET/CT image characteristics of a patient with splenic PSCS.

## Case report

**Figure 1.**
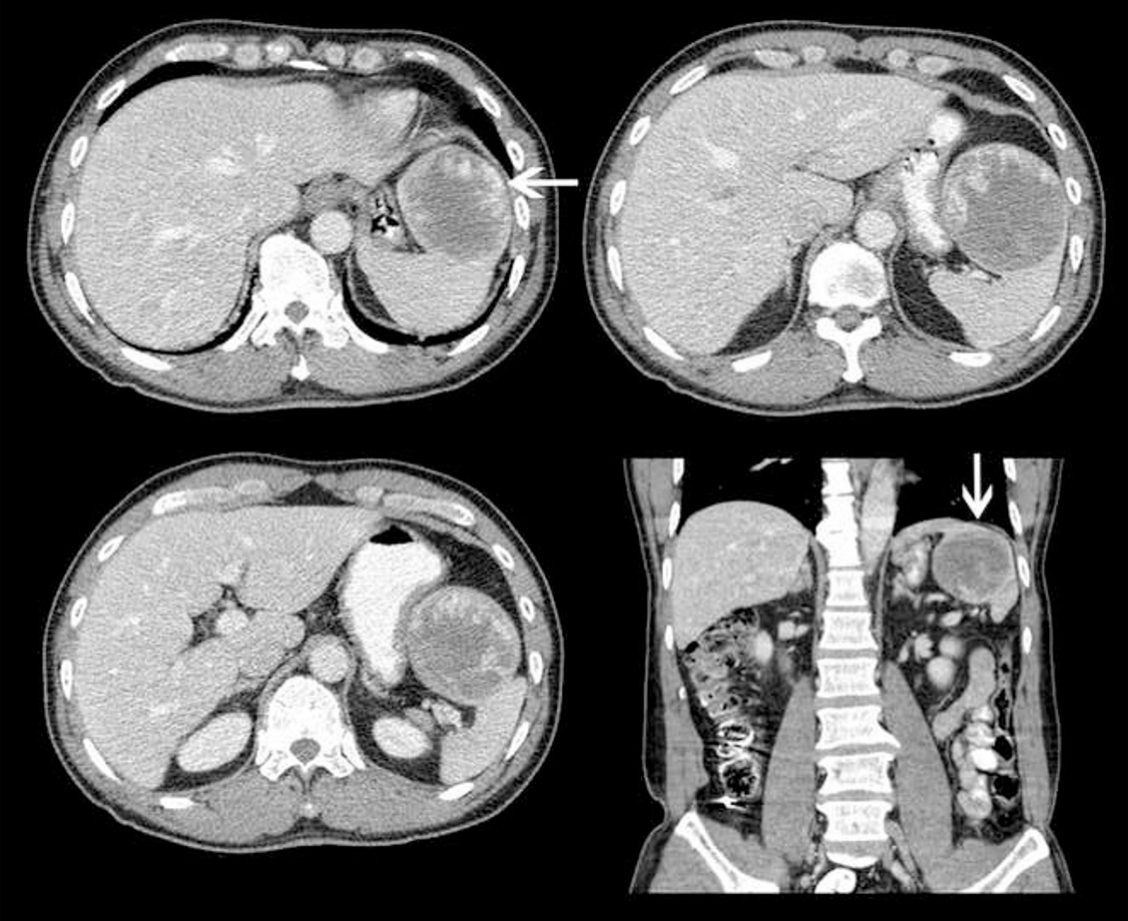
Transaxial and coronal images of the splenic mass at presentation, measuring 7.5 × 7.3 × 7.0 cm (AP × ML × CC) and showing a well-defined predominantly hypoattenuating mass with a rim of peripheral serpiginous enhancement (arrows).

**Figure 2.**
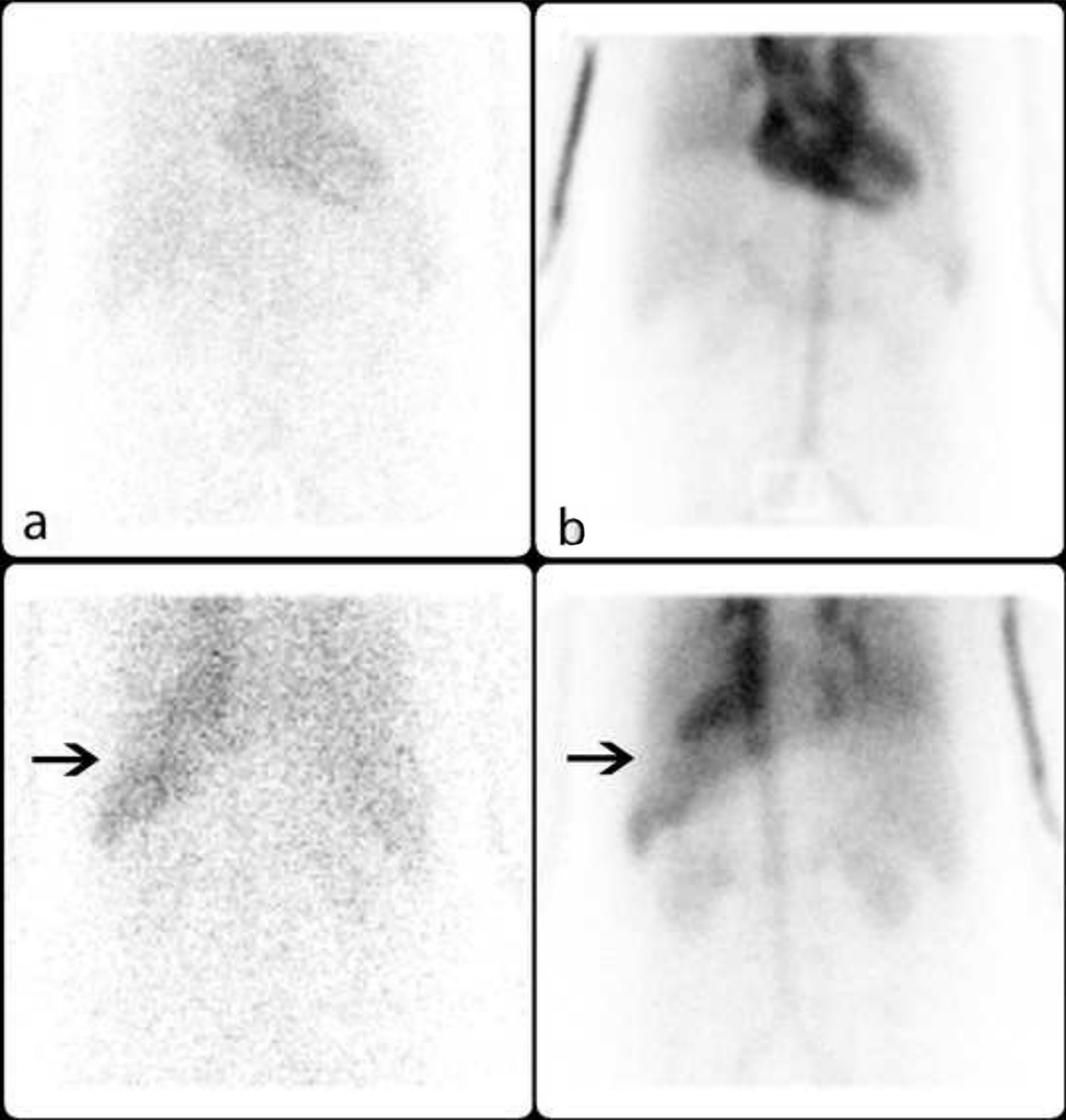
(a) Anterior and posterior blood flow images of ^99m^Tc-RBC scan showed a normal blood flow to the spleen. (b) Delayed images showed a photopenic defect in the region of the splenic mass with mild peripheral blood pooling (arrows).

**Figure 3.**
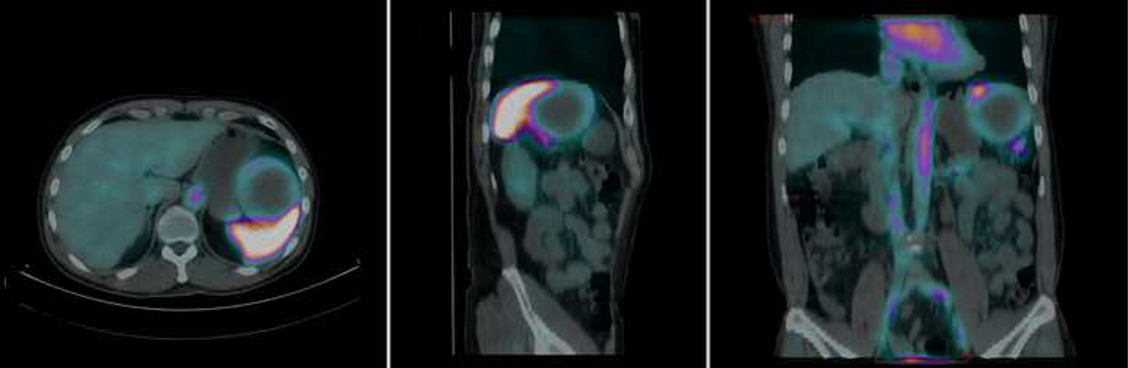
^99m^Tc-RBC scan SPECT/CT fusion images, transaxial, sagittal and coronal of the splenic mass.

A 63-year-old male presented with abdominal pain and was diagnosed with a 7.5 × 7.3 × 7.0 cm (AP × ML  × CC) splenic mass on CT. It was predominantly hypo-attenuating with peripheral serpiginous enhancement. The lesion was very well defined and the imaging characteristics were interpreted as being consistent with a benign mass such as haemangioma or hamartoma; however, malignancy could not be excluded (Figure 1). A three-phase ^99m^Tc-RBC scan was performed to further characterize the splenic mass. It showed a normal blood flow and on delayed images showed a photopenic defect at the location of the mass with mild peripheral blood pooling (Figures 2 and 3), which was interpreted as possibly representing a haemangioma with central thrombosis, or an atypical haemangioma, however malignancy could not be excluded and further imaging was recommended.

**Figure 4.**
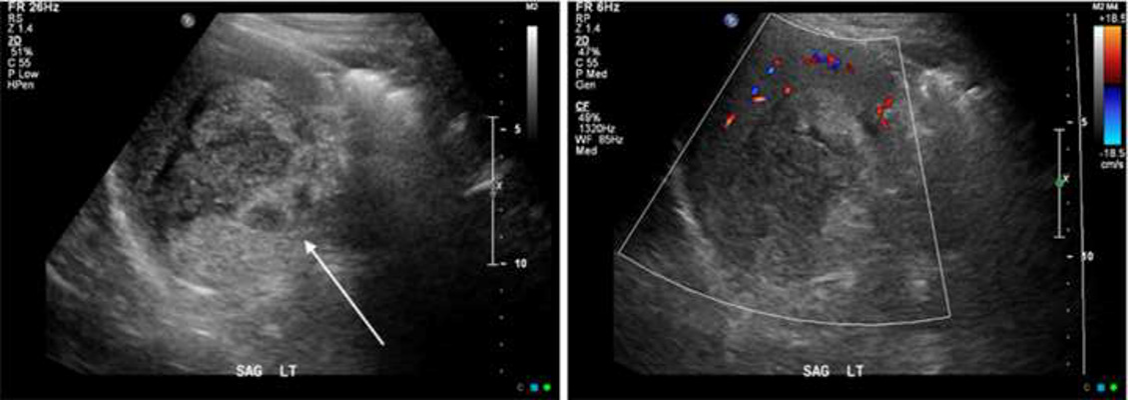
Ultrasound images showed a hypoechoic splenic mass with no internal vascularity (arrow). There were no focal lesions in the liver.

Since the patient was well clinically, the splenic mass was followed conservatively with serial ultrasounds. An initial ultrasound showed a heterogeneous, mostly hypoechoic mass with no internal vascularity and no focal lesions in the liver. The findings on the ultrasound study were interpreted as being in keeping with haemangioma (Figure 4). A follow-up ultrasound performed 10 months later showed that the splenic mass was stable in size and was still likely a haemangioma. Sixteen months after the initial CT, however, the patient presented with a 2-month history of 35 lb weight loss, failure to thrive, 1 month of daily diarrhea, fever and drenching night sweats, bloating, distension and decreased appetite. His haemoglobin was 75 g l^–1^ (normal 120–160 g l^–1^), and platelets 9 (normal 140–450 10^9^ l^–1^). A follow-up ultrasound showed an increase in the size of the splenic mass and new liver lesions.

**Figure 5.**
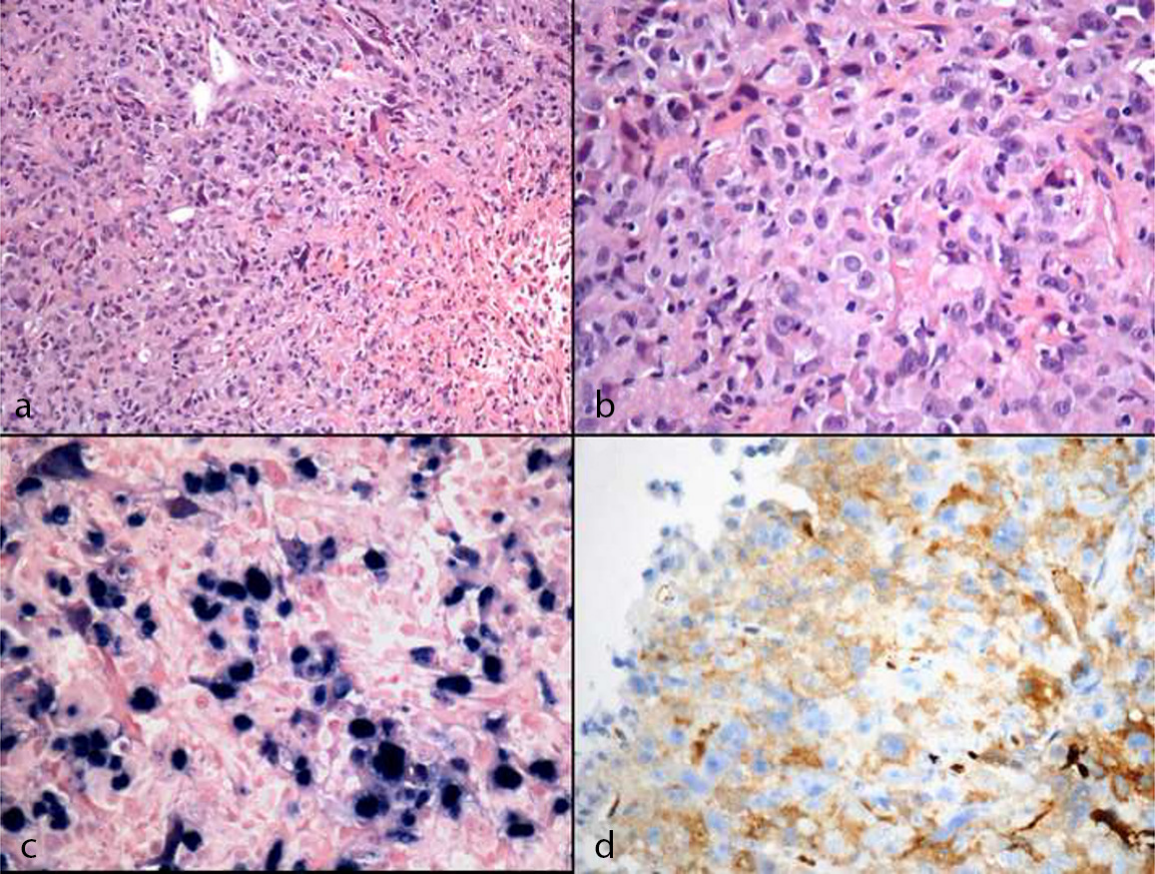
Core biopsy of the liver. (a) H/E staining (OM 200×), (b) H/E staining (OM 400×), (c) EBER positive staining (OM 400×) and (d) fascin positive staining (OM 400×).

A core biopsy of the liver showed polymorphic atypical proliferation of poorly differentiated cells associated with coagulated necrosis and a sprinkling of small lymphocytes with eosinophils. These pleomorphic cells included large multinucleated forms with open vesicular chromatin and prominent eosinophilic nucleoli. Immunohistochemistry was positive for EBER and Fascin and negative for CD21, CD35, CAM 5.2, CD31, ERG, S100, pan-keratin, CD45, CD43, CD34, ALK-1, PAX 5, CD30, CD68, CD23, HMB-45, lysozyme, myeloperoxidase, podoplanin, CD20 and muscle specific A (Figure 5). These findings were consistent with pleomorphic spindle cell sarcoma (PSCS, previously known as malignant fibrous histiocytoma, MFH). A bone marrow biopsy was negative.

**Figure 6.**
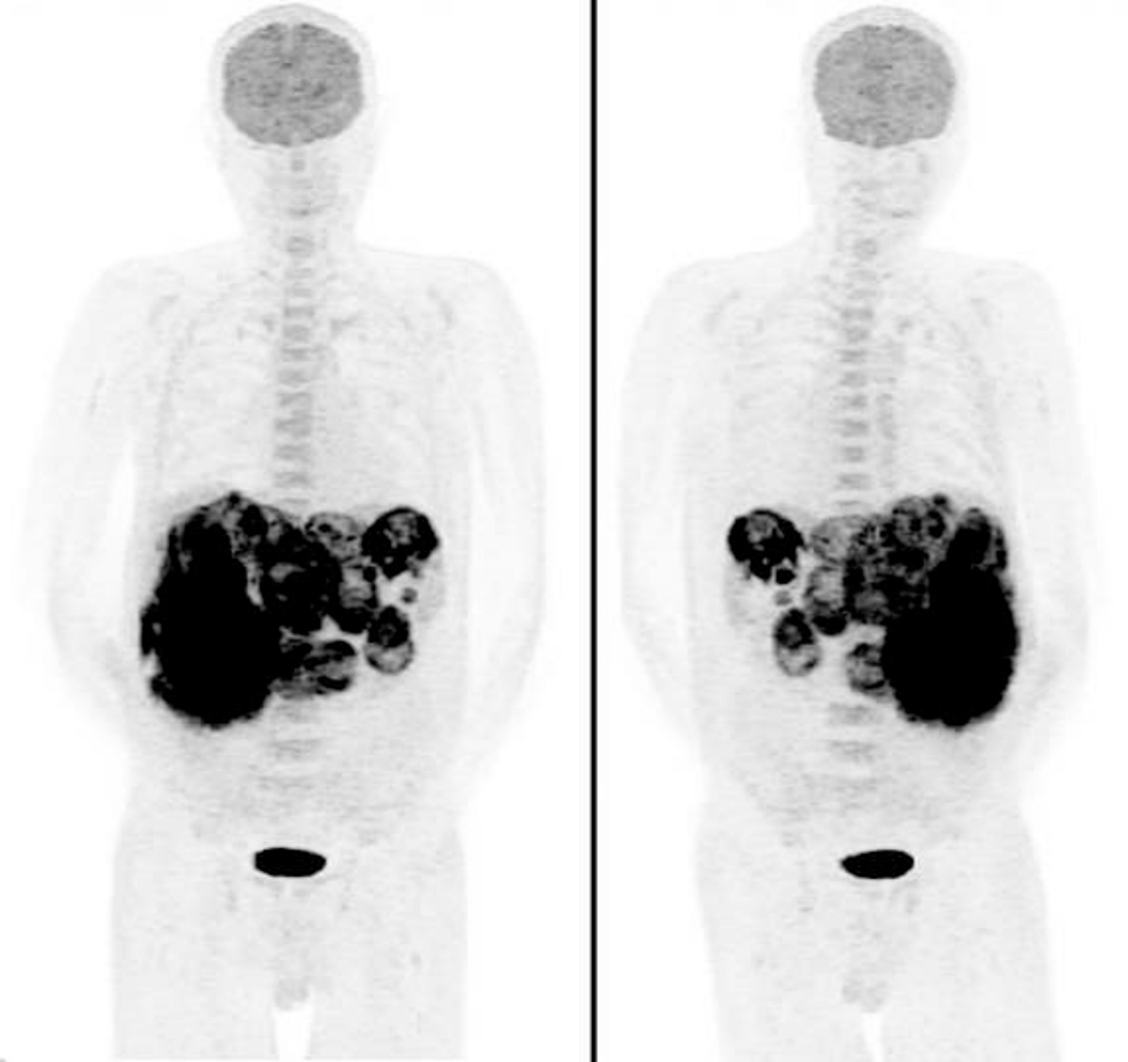
A staging ^18^F-FDG PET/CT. Maximum intensity projection (MIP) images showed innumerable intensely ^18^F-FDG avid lesions in the liver and spleen.

**Figure 7.**
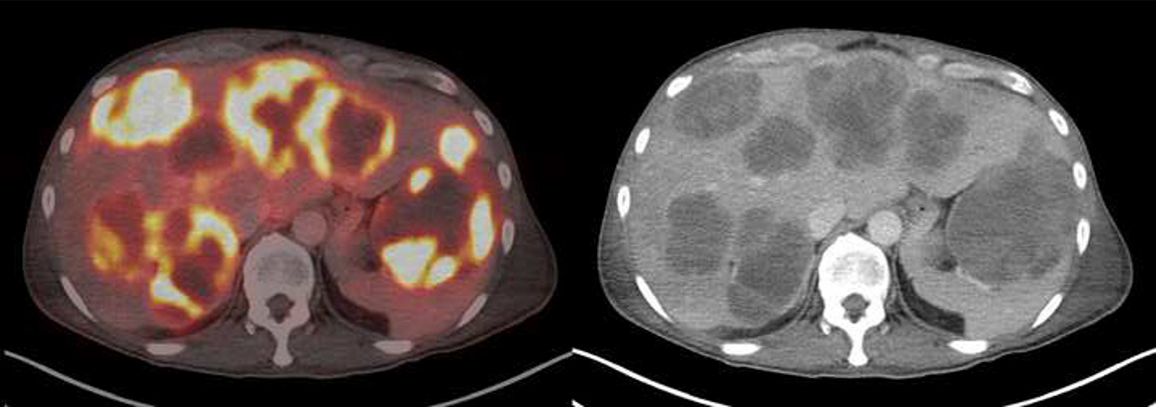
Transaxial PET/CT fusion (left) and CT (right) images showed that the largest splenic mass measured 9.6 × 7.5 cm with maximum standardized uptake value (SUV_max_) 15.8. The liver and splenic lesions were almost entirely necrotic, with a thin rim of intense ^18^F-FDG uptake.

The patient was referred for an ^18^F-FDG PET/CT for staging. Maximum intensity projection (MIP) images showed innumerable intensely ^18^F-FDG avid lesions in the liver and spleen (Figure 6). The largest splenic mass measured 9.6 × 7.5 cm with maximum standardized uptake value (SUV_max_) 15.8 (Figure 7). The lesions were almost entirely necrotic, with a thin rim of intense ^18^F-FDG uptake. The patient passed away a few days later, before any treatment plan could be initiated.

## Discussion

Haemangiomas are the most common benign tumours of the spleen. They are usually intrasplenic, non-capsulated, solitary, and most measure less than 2 cm.^1^ Splenic haemangiomas have been described to show delayed-phase contrast enhancement in a mottled pattern rather than a centripetal fill-in pattern which is commonly seen in liver haemangiomas.^2^ Our patient’s findings were not diagnostic for a typical haemangioma but were more in keeping with an atypical haemangioma, with a differential diagnosis including other benign processes such as a hamartoma, splenic abscess or malignancies such as haemangiosarcoma, leiomyosarcoma, fibrosarcoma, cystadenocarcinoma, teratoma or metastases.^1^

The Tc-99m labelled RBC scans of haemangiomas show a photopenic area in the region of the haemangioma on perfusion and early blood pool images with filling-in on delayed images and mildly increased uptake relative to surrounding splenic tissue.^3^ Our patient’s three-phase ^99m^Tc-RBC scan showed a photopenic defect on delayed images with mild peripheral blood pooling, which was not in keeping with a typical haemangioma and could have represented an atypical haemangioma or other benign or malignant lesions. Our patient’s ultrasound findings were also non-specific and while they can be seen in haemangiomas, they can also be seen in other benign or malignant splenic lesions.^1^ Since the patient was well clinically and the imaging findings were suggestive of an atypical haemangioma, a conservative approach was taken and the patient was followed with serial ultrasound imaging, which showed stability of the splenic lesion for an initial 12 months. Unfortunately, the patient then developed new and severe symptoms and deteriorated rapidly clinically.

“Pleomorphic spindle cell sarcoma” was previously known as “malignant fibrous histiocytoma” and comprises a heterogeneous group of tumours without a specific known line of differentiation. While these tumours are now believed to be the most frequently encountered malignant soft tissue tumours,^4,5^ they are extremely rare in the spleen, with less than 20 cases of splenic MFH reported in the literature.^6–9^ Splenic MFH occurs in adults aged 40–60 with a slight male predominance, and symptoms such as abdominal pain, splenomegaly, weight loss and fever are common in about 70% of these patients. It is a very aggressive malignancy with high potential of local recurrence and distant metastases, usually to the liver.^10^

^18^F-FDG PET/CT has been shown to have a potential role in differentiating malignant splenic lesions from common benign splenic lesions, as well as in staging malignant splenic lesions; however, false positives such as inflammatory or infectious processes (*i.e.* granuloma, abscess) and false negatives, such as metastases from poorly ^18^F-FDG avid malignancies like renal cell carcinoma, can complicate PET/CT image interpretation.^11^

^18^F-FDG PET/CT has proven to be useful in the staging of MFH and identification of metastatic disease in unusual locations^12–14^ and the use of ^18^F-FDG PET/CT for staging of aggressive sarcomas including MFH is recommended in the joint RCR and RCP evidence-based guidelines for the use of PET/CT in the United Kingdom (2016);^15^ however, to our knowledge, the use of ^18^F-FDG PET/CT in the staging of splenic PSCS has not been previously described. Owing to the highly aggressive nature of this splenic malignancy, including two recent reports of spontaneous tumour rupture^6,10^ timely imaging with PET/CT is crucial for implementation of treatment. Histopathological examination is essential both for confirming the diagnosis of splenic PSCS and for grading and staging of these tumours. The mainstay of therapy for all PSCS tumours is surgical resection, although in this case the extent of the disease ruled out a surgical treatment option.^5^ The standard chemotherapy for PSCS includes agents, such as doxorubicin or ifosfamide; however, the literature suggests that chemotherapy does not improve overall survival for PSCS patients as a whole, although certain subpopulations such as patients with PSCS in the extremity, can show improved overall survival with chemotherapy.^16^

## Learning points

1. Pleomorphic spindle cell sarcoma (PSCS) was previously known as malignant fibrous histiocytoma (MFH), and are now believed to be the most frequently encountered malignant soft tissue tumours, although they are very rare in the spleen.
2. PSCS of the spleen can mimic a benign lesion such as an atypical haemangioma on CT, ultrasound or ^99m^Tc-RBC scan.
3. Splenic PSCS can be staged with ^18^F-FDG PET/CT to determine the full extent of the disease and 2016 UK PET/CT guidelines recommend the use of ^18^F-FDG PET/CT in the staging of aggressive sarcomas including MFH.
4. Owing to the highly aggressive nature of splenic PSCS, timely imaging with ^18^F-FDG PET/CT is crucial for implementation of treatments such as surgical excision or chemotherapy.

## Consent

Informed consent to publish this case (including images and data) was obtained and is held on record.
